# Efficient gene expression signature for a breast cancer immuno-subtype

**DOI:** 10.1371/journal.pone.0245215

**Published:** 2021-01-12

**Authors:** Ben Galili, Xavier Tekpli, Vessela N. Kristensen, Zohar Yakhini

**Affiliations:** 1 Computer Science Department, Technion – Israel Institute of Technology, Haifa, Israel; 2 Department of Cancer Genetics, Institute for Cancer Research, Oslo University Hospital, Oslo, Norway; 3 Arazi School of Computer Science, Interdisciplinary Center, Herzliya, Israel; University of California Davis, UNITED STATES

## Abstract

**Motivation and background:**

The patient’s immune system plays an important role in cancer pathogenesis, prognosis and susceptibility to treatment. Recent work introduced an immune related breast cancer. This subtyping is based on the expression profiles of the tumor samples. Specifically, one study showed that analyzing 658 genes can lead to a signature for subtyping tumors. Furthermore, this classification is independent of other known molecular and clinical breast cancer subtyping. Finally, that study shows that the suggested subtyping has significant prognostic implications.

**Results:**

In this work we develop an efficient signature associated with survival in breast cancer. We begin by developing a more efficient signature for the above-mentioned breast cancer immune-based subtyping. This signature represents better performance with a set of 579 genes that obtains an improved Area Under Curve (AUC). We then determine a set of 193 genes and an associated classification rule that yield subtypes with a much stronger statistically significant (log rank p-value < 2 × 10^−4^ in a test cohort) difference in survival. To obtain these improved results we develop a feature selection process that matches the high-dimensionality character of the data and the dual performance objectives, driven by survival and anchored by the literature subtyping.

## Introduction

Molecular profiling of tumors has improved disease management and personalized cancer medicine. In particular, gene expression-based signatures distinguish five breast cancer subtypes [1–3]—Luminal A, Luminal B, Her2-enriched, Basal-like and Normal-like. These subtypes are clinically relevant for breast cancer management and personalized treatment [4]. The tumor microenvironment influences cancer pathogenesis, progression, prognosis [5, 6] and disease characteristics [7]. In breast cancer, high immune infiltration has been associated with better clinical outcome [8, 9]. In particular, high CD8+ T cell counts are associated with better overall survival in estrogen receptor (ER) negative patients [10, 11]. In addition, high immune infiltration has been associated with an increased response to neo-adjuvant and adjuvant chemotherapy [12]. Understanding the effect of the immune landscape of tumors and their environment can lead to better characterization and subtyping in breast cancer. Such insight could possibly contribute significantly to improving disease management. As immunotherapies emerge as a new treatment option for breast cancer, it is important to determine which patients will benefit from such treatment alternatives.

A global approach to understanding the role of the tumor’s immune context in breast cancer was taken by several recent studies [13–15]. In [15], the authors introduce a clinically relevant immune associated classification of breast cancer samples. The study covers 15 breast cancer cohorts, spanning 6101 breast cancer samples, and reports a gene expression-based signature that supports a classification with a statistically significant difference in prognosis. In principle, tumors with an intermediate level of immune infiltration were found to have a worse prognosis independently of other known prognostic clinicopathological features, such as the Prediction Analysis of Microarray 50 (PAM50) molecular subtyping. Deriving a gene expression signature associated with, but not identical to, immune infiltration, however, leads to even stronger prognostic indications. The immune-associated classification of [15] was developed over 658 genes. In this work, we develop a more efficient signature in terms of the following aspects. First, we determine a set of 573 genes that represent the molecular clustering, as presented in [15], with better performance (both AUC and Cluster B recall). This set of genes is reported in Point A gene list in S1 Text. Second, we establish a set of 193 genes and an associated classification rule that yield subtypes with a much stronger statistically significant (log rank p-value <2 × 10^−4^ in a test cohort) difference in survival. This set of genes is reported in Point B gene list in S1 Text. Here, as well as in all other relevant points in the manuscript, the word survival delineates overall survival. In particular, this study does not address a signature that would be indicative of the response to any particular therapy. The work flow described here deals with an optimization task for a dual performance metric: accuracy with respect to the immune subtype in [15] and statistical significance with respect to prognosis. To obtain the aforementioned improved results, we developed a feature selection process that matches the high-dimensionality character of the data and the dual performance requirements. The methods described herein can, therefore, be generalized to similar feature selection tasks.

In particular, we anchor the first phase of the feature selection to the immune subtype classification and then focus further on prognosis. The iterative approach we describe herein allows the number of features needed to predict a particular trait to be reduced while monitoring the specificity and sensitivity of the prediction as well as the effect on prognostic differences. The last step of our process involves developing a classification based on the relative expression levels of the genes in the proposed signature. This last step is of particular importance in making our findings potentially useful in the clinic.

To summarize, we develop an efficient expression signature associated with survival in breast cancer. We describe a method for developing this signature that is also applicable in similar cases. The method selects features that lead to significant association to survival while also respecting a previously known classification.

## Materials and methods

Our approach to the feature selection task has four components. The first is a filtering step. The second is based on a logistic regression step with gene sets decreasing in size, attempting a classification into Cluster B vs. Not Cluster B. Cluster B is the middle immune cluster defined by [15]. The third step involves using the validation data to select the best set of genes from this feature selection process. This third step is either driven by the classification accuracy or by performance in a survival statistical test, applied to the validation data. Finally, we take the set of genes obtained through the above process and develop a rank-based classifier that maps a profile developed using these genes to generate a prognostic prediction. This last step demonstrates the feasibility of translating the classification mechanism to a clinical set-up that is not dependent on the training cohorts. We now describe in detail the above steps.

### Data

We used 13 of the 15 cohorts used in [15]. We designate Cohorts MAINZ (*n* = 200), STAM (*n* = 856) and UPSA (*n* = 289) as test cohorts (this is the same as in [15]). We use the remaining 10 cohorts as training cohorts, for a total of 4546 samples with 9461 genes. All the gene expressions in all the cohorts are normalized and have a distribution of *N*(0, 1). When driving our selection by classification accuracy, we use 20% as a validation set for determining the final selected set of genes. When driving our selection by log rank performance, we combine 50% of the test cohorts to serve as a validation set. The other half serves as test data for the log rank performance. We now describe, in detail, the three stages of our selection process:

1. Wilcoxon rank-sum (WRS) filtering.
2. Iterative logistic regression.
3. Select the best set of genes according to the validation set—first using normalized expression and second, based on relative expression.

### WRS filtering

Our first step is to run a WRS test [16, 17] as a filter method. The selection of the genes in this step is independent of any machine learning algorithm. We filter out genes that are not differentially expressed with respect to Cluster B vs. Not Cluster B, as defined in [15]. We use p-value = 0.1 as our threshold and each gene with a WRS p-value > 0.1 is filtered out. After the WRS filtering, we are left with 6557 genes, out of the initial 9461 genes. This point represents a False Discovery Rate (FDR) [18] of 0.14.

### Iterative lasso logistic regression

Iterative approaches, such as Orthogonal Matching Pursuit (OMP—[19]), have proven to be efficient in feature selection processes. Using the OMP approach, we construct the set of features iteratively, working with z-score normalized feature values. Each iteration consists of running a regression algorithm and selecting the feature with the highest weight. The process is initialized with the target vector $\vec{y}$ and, in each iteration, the target is updated to the residual. Since the features are normalized, the weights of the solution vector are associated with the impact of each feature on the predicted variable. The greater the weight (absolute value), the greater the effect on the prediction value. We use this property to drive an iterative lasso backward selection algorithm. We note that applying forward selection in our data yielded far inferior results and the backward approach certainly led to better performance. Specifically, forward selection produced a validation accuracy of 0.8 while our iterative lasso backward selection algorithm generated a validation accuracy of 0.91.

Another approach is the Relaxed Lasso [20], where the lasso regression is executed twice with different regularization factors. [20] demonstrates, using theoretical and numerical evidence, that Relaxed Lasso produces sparser models with equal or lower prediction losses than the regular lasso estimator for high-dimensional data.

Our approach is an iterative one that starts with all features and rejects features as we move through a while loop. In each iteration we run a logistic regression penalized by lasso (λ = 1) to calculate the weight for each gene. Then, we remove genes with low weight (absolute value). In the first iterations, we remove 10% of the genes with the lowest weights (absolute value). From 900 genes downwards, we remove only one gene in each iteration. The pseudocode is given in Algorithm 1.

**Algorithm 1**: Pseudocode of the algorithm we use for selecting an efficient set of genes, where *LR*(*D*, Ω) is the logistic regression algorithm (lasso in our case) and $nnz(\vec{w})$ is the number of non-zero elements in $\vec{w}$. The function *argsort* sorts the indices of an array according to the values it contains.

**Iterative logistic regression gene set selection**

**input**: Normalized gene expression dataset—*D*

**output**: Sparse gene expression dataset

initialization

Ω = {set of all genes}

$\vec{w}\neq 0$

**while** $nnz(\vec{w})>900$ **do**

 $\vec{w}=LR\left(D,\Omega \right)$

 // find the indices of 10% smallest absolute values

 sorted_index_w = *argsort*(|*w*|)

 10%_index = 0.1 × *size*(*w*)

 smallest_set = sorted_index_w[0:10%_index]

 Ω = Ω−smallest_set

**end**

**while** $nnz(\vec{w})>50$ **do**

 $\vec{w}=LR\left(D,\Omega \right)$

 smallest_w_index = *argmin*(|*w*|)

 Ω = Ω−smallest_w_index

**end**

**return** Ω

### Selection based on classification accuracy

**Fig 1:**
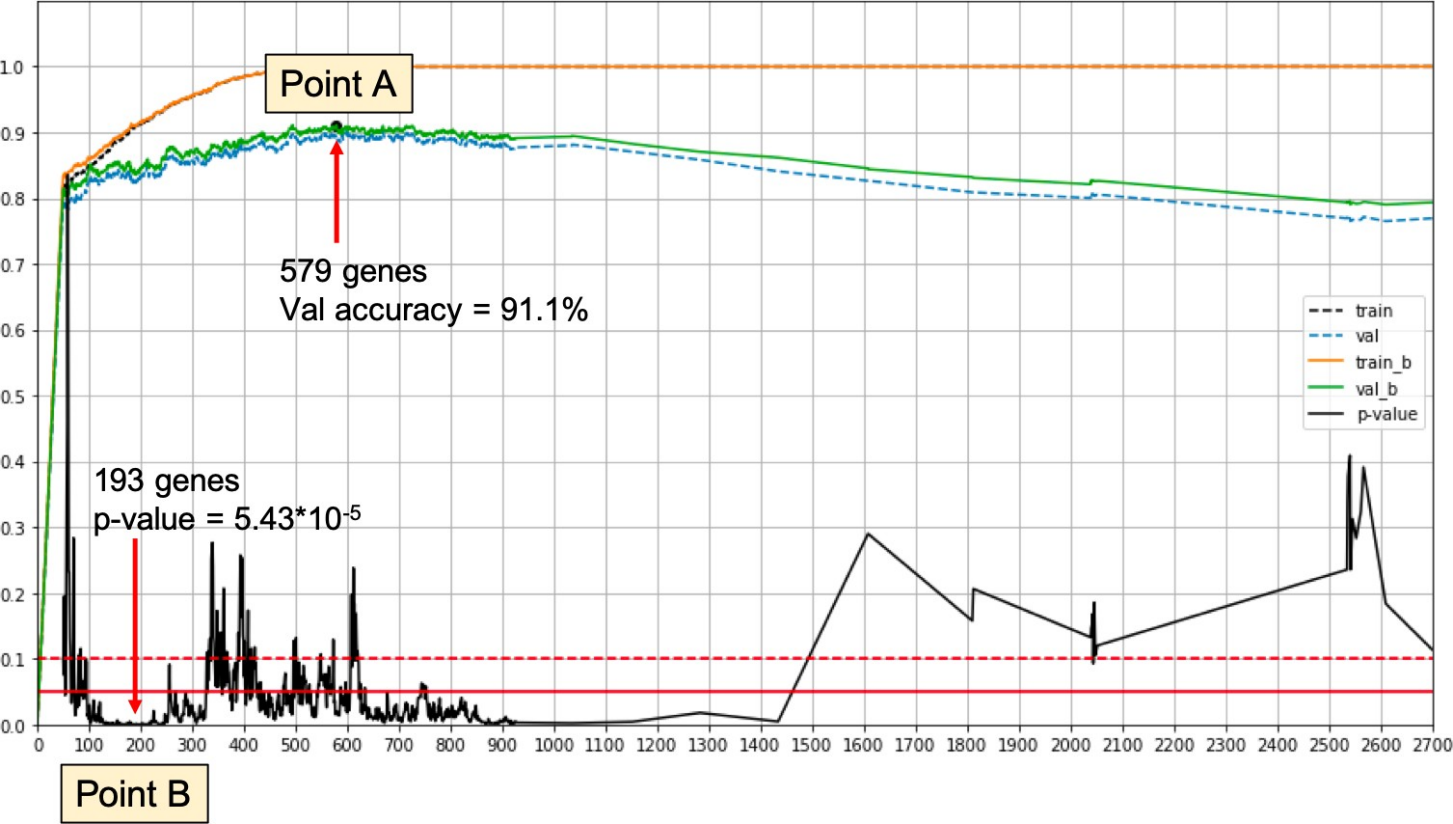
Overview illustration of the gene selection process. Point A—Best validation accuracy for the classification driven process. Point B—Best log rank p-value on a validation set. The signature of Point B achieves log rank p-value < 2 × 10^−4^ and log rank p-value < 2 × 10^−3^ when using only relative expression values.

We combine our iterative approach with a training/validation split. In each iteration, we calculate the validation accuracy. Classifiers are trained with a three-way loss function for clusters A, B and C. For the validation we evaluate Cluster B vs. Not Cluster B. We select the best performing set of genes. This process yields Point A in Fig 1. The set of genes thus obtained is called Γ_*A*_.

### Selection based on log rank performance

In this process we incorporate the log rank test into the training phase. We take the test cohorts with 1300 samples and split them into validation and test sets. We use the new validation set in the iterative process to evaluate the performance after each iteration. In each iteration we predict the label of all validation samples and then conduct a log rank test comparing the samples called Cluster B to those called Not Cluster B. We choose the set of features with the smallest log rank p-value. This process yields Point B in Fig 1. The set of genes thus obtained is called Γ_*B*_.

### A signature based on relative expression

In practice, results derived from the normalized data are not directly useful in working with new samples. When diagnosing a single patient, no normalization context can be assumed—beyond that which is internal to the patient’s sample. To enable practical use of the genes in Γ_*B*_, we trained a new classifier based on their relative expression as derived from the raw data that underlies the samples in our training set. The idea is that the relative expression levels of these genes can be computed in the context of any signature sample, without the need to normalize it back to the context of the training cohorts. For this purpose, consider the raw expression data instance *E*(*g*, *d*) measured for every gene *g* and a single sample *d*. We define a relative expression value *Q*(*g*, *d*) for every *g* ∈ Γ_*B*_ and every training sample *d*. First we sort *E*(*g*, *d*) to get the ranks 1 ≤ *R*(*g*, *d*) ≤ *N* = |Γ_*B*_| (= 193). Now define:

$$\begin{array}{c} Q\left(g,d\right)=\lceil \frac{10\cdot R(g,d)}{N}\rceil \end{array}$$ (1)

In other words, we map every gene to its relative decile within the set Γ_*B*_, in the context of sample *d*.

We now train a Naive Bayes classifier, based on the features *Q*(*g*, *d*), to classify the samples into two prognostic subclasses: *GPx* = *Not*_*Cluster*_*B* and *BPx* = *Cluster*_*B*. To classify a new sample (which has not been seen in the training process), we just transform all gene expression levels there, for all *g* ∈ Γ_*B*_, to relative levels (rank deciles) as above and execute the trained classifier. Note that 10 is obviously a hyperparameter of this process.

### Kaplan Meier curves

The Kaplan Meier curves in this paper were generated using the Python library *lifelines*. They include the 0.95 confidence envelope and an indication of the Q3 level of survival for each class with corresponding colors.

## Results and discussion

### Selected efficient signatures

The two feature selection approaches described in the Methods section resulted in two gene sets with the best observed performance:

1. 579 genes for the classification accuracy driven approach; designated as Point A in Fig 1.
2. 193 genes for the log rank performance driven approach; designated as Point B in Fig 1.

Note that when using different training validation set splits, we may get different results. This point is addressed below. An overview of the selection process, as run on our data, is depicted in Fig 1.

### Improved Cluster B classification with 579 genes

We compared our results to [15]. As shown in Table 1, we improved the classifier recall and the AUC. We also improved the log rank p-value slightly. We note that we also achieved a modest advance in the number of genes that support the signature.

**Table 1:** Performance overview—Point A.

	This work	Tekpli et al.
# genes	579	658
Cluster B recall	0.89	0.69
AUC	95.4%	85.8%
Log rank p-value	0.00018	0.00027

**Fig 2:**
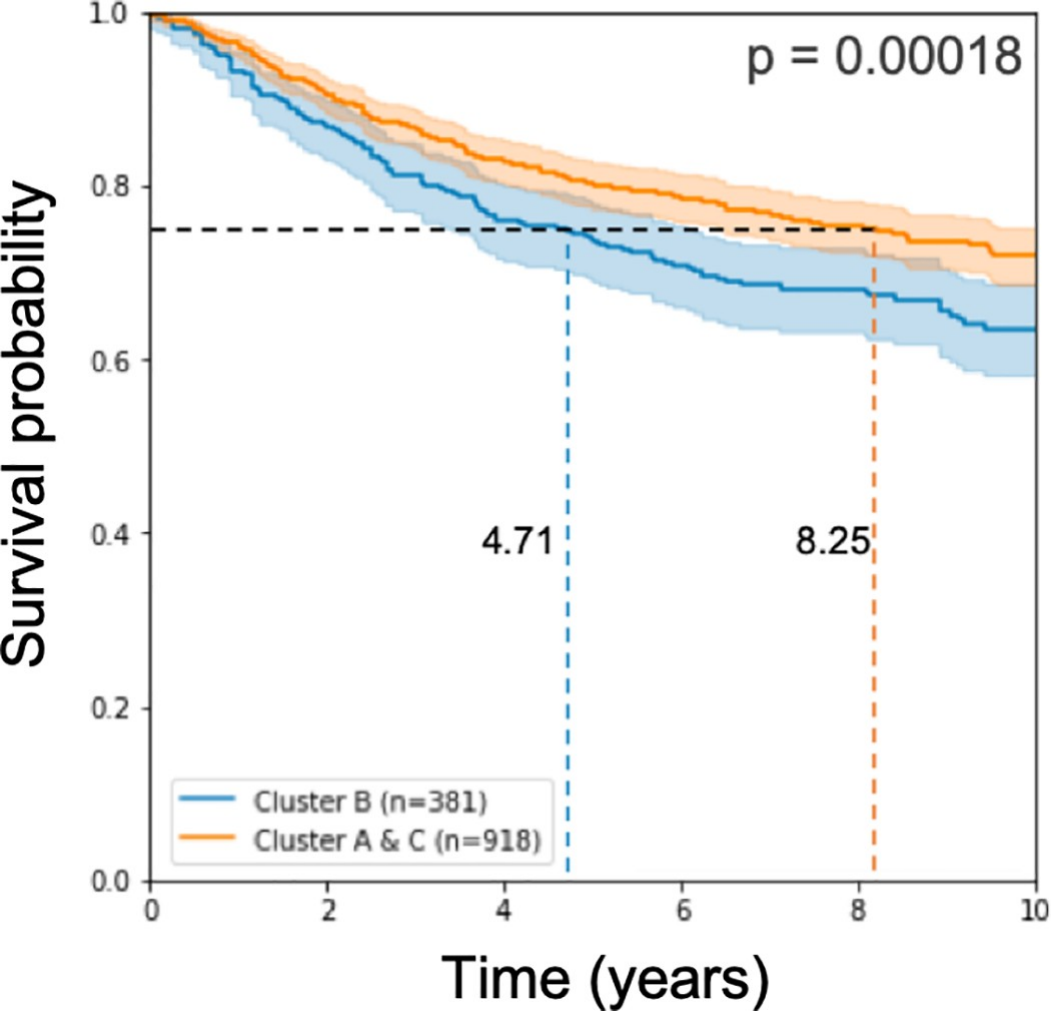
Kaplan Meier curve and log rank p-value on the test cohorts. The model used 579 genes for learning.

Working with these 579 signature genes that we found, we classified the test samples and applied log-rank statistical analysis on the results of the classification. We observe, as in [15], a prognostic disadvantage to the samples called Cluster B (see Fig 2).

### Improved prognostic signature with 193 genes

Reducing the number of selected genes reduces the classifier’s performance as well, as we can see in Table 2. The procedure of feature selection for better prognosis is a new task and will be further addressed below. Although the classifier’s performance decreased, the log rank p-value stayed the same and the number of selected genes dropped to 193.

**Table 2:** Performance overview—Point B.

	This work	Tekpli et al.
# genes	193	658
Cluster B recall	0.71	0.69
AUC	89.4%	85.8%
Log rank p-value	0.00017	0.00027

**Fig 3:**
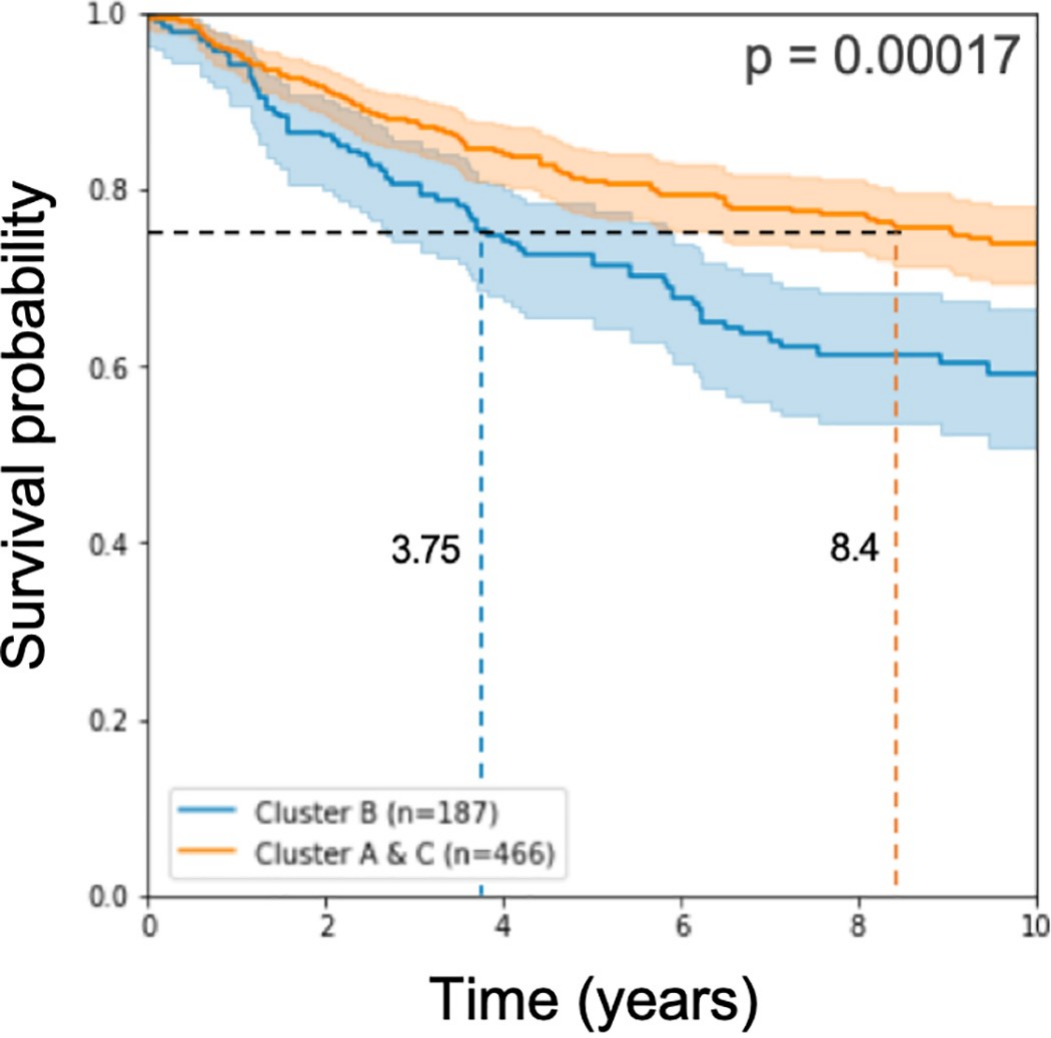
Kaplan Meier curve and log rank p-value on half of the test cohorts. The model used 193 genes for learning.

Working with these 193 signature genes that we found, we classified the half of the test samples we left for testing and applied log-rank statistical analysis on the results of the classification. As in approach 1 above, the prognostic disadvantage to the samples called Cluster B remains significant (see Fig 3).

### A signature based on relative expression yields accurate prognostic predictions

**Fig 4:**
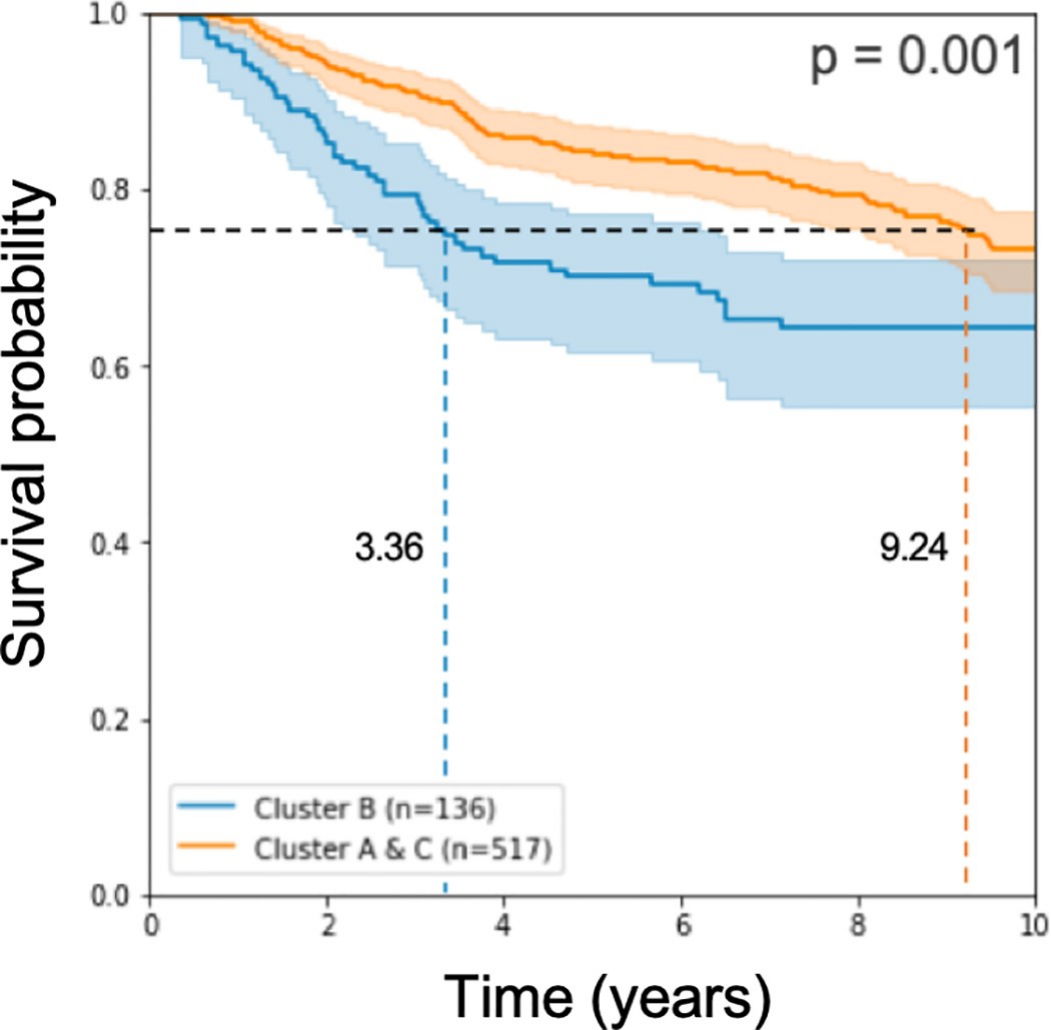
Kaplan Meier curve and log rank p-value on half of the test cohorts. The model used expression rank values from 193 genes for learning.

As described in the Methods section, we applied a Naive Bayes approach to classify test patients according to their predicted prognosis: *GPx* (good prognosis) and *BPx* (bad prognosis). Survival analysis results in a log rank p-value of 10^−3^ when computed for *n* = 653 test samples (see Fig 4).

### Trained classifier results are associated with tumor infiltrating lymphocytes levels

**Fig 5:**
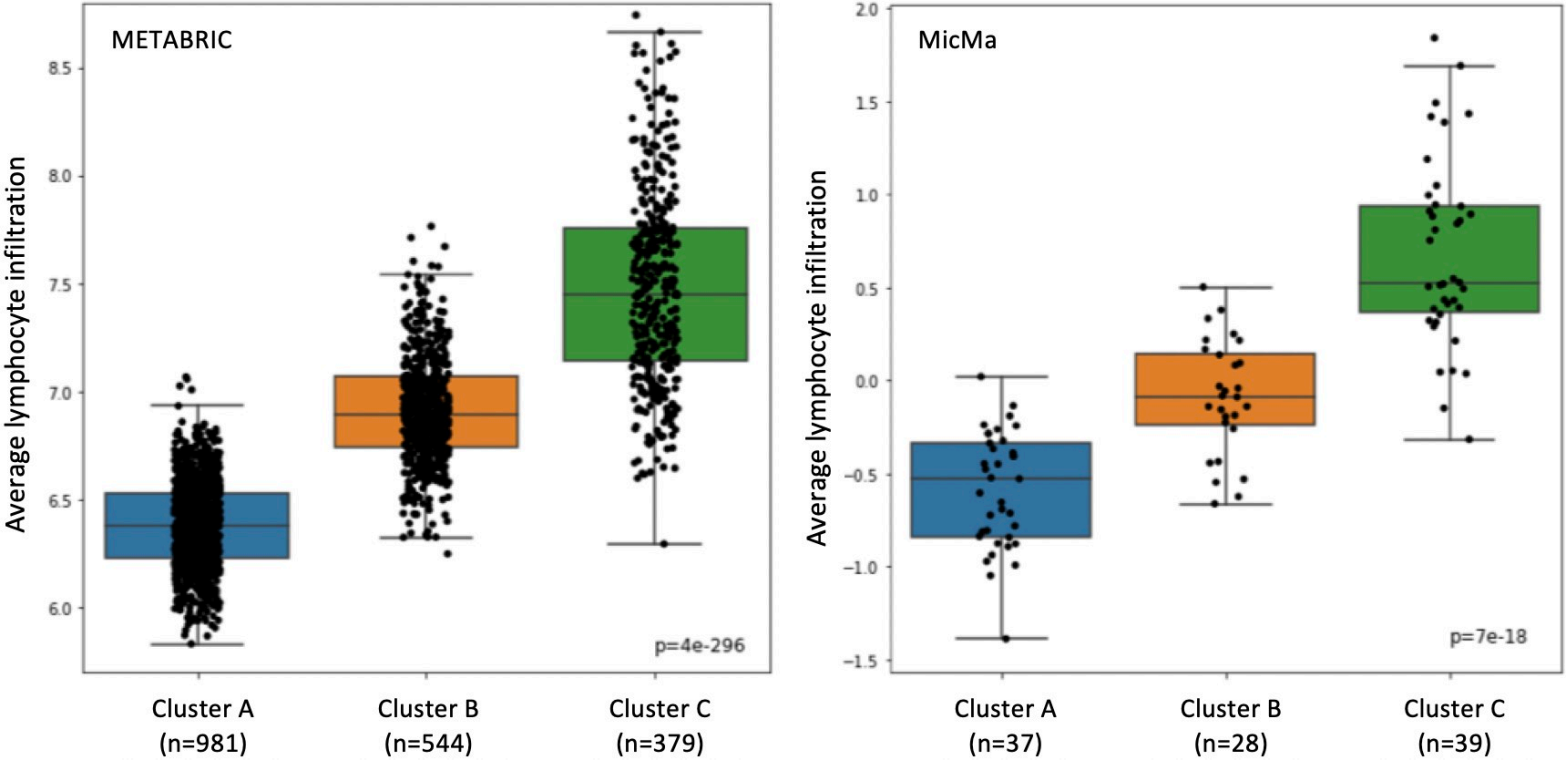
Lymphoid score differences. Lymphoid scores are represented in box-plots according to the training predictions with the selected 193 genes. On the left is the Metabric cohort and on the right, the MicMa cohort.

We further examined our classification results on the training set, using the 193 gene set (Point B). We checked that the association between the clusters and the lymphocyte infiltration did not break. As we can see in Fig 5, the lymphocyte infiltration is increasing when moving from Cluster A to Cluster B to Cluster C. We also tested the significance of the classes using the Kruskal–Wallis test, as in [15], and got a value of 4 × 10^−296^ & 7 × 10^−18^ for the Metabric and MicMa cohorts, respectively. These results are similar to these of [15].

### Enrichment analysis

**Fig 6:**
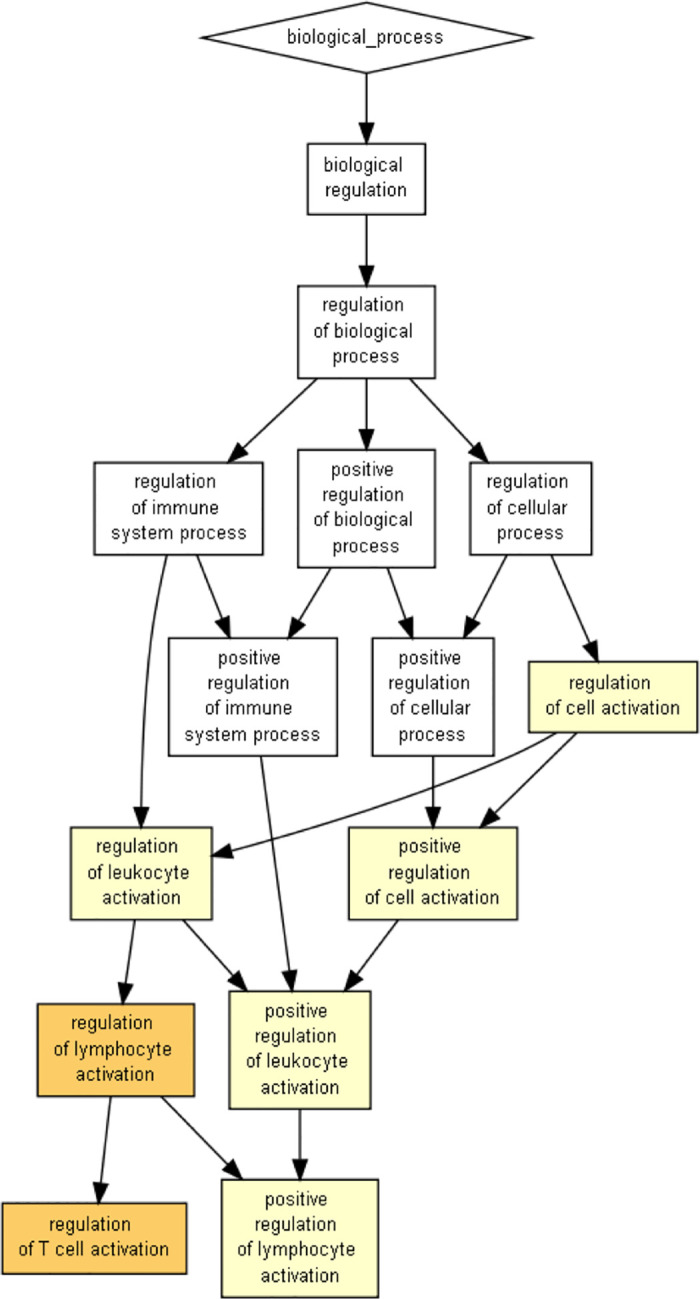
Gene Ontology biological processes statistically enriched in the selected 193 genes, using GOrilla [21, 22]. Color intensity represents the statistical significance of the enrichment.

We used the GOrilla (Gene Ontology enRIchment anaLysis and visuaLizAtion) [21, 22] tool to examine statistical enrichment of GO (Gene Ontology) annotations. As shown in Fig 6, our selected set of 193 genes is significantly enriched with immune system genes.

### Characteristics of *BPx*

To understand more deeply our findings, we examined the *BPx* class.

First, we analyzed the composition of *BPx*, in terms of known breast cancer subtypes and other patient characteristics. The results are described in S1 Fig in S1 Text. The properties analyzed include age, tumor grade, tumor stage, laterality, lymph node metastasis number, ER status and others. In summary, we cannot reject the no difference null hypothesis for any subset of the tested properties at FDR = 0.05. The composition of *BPx*, in terms of PAM50 subtypes, is further addressed below.

Second, we compared *BPx* to [15]’s Immune Cluster B (CB) in the combined cohort of 4546 patients. We observed 447 patients in the symmetric difference *CB*Δ*BPx*. Of these, we observed 268 patients in CB\*BPx* and 179 patients in *BPx*\CB. Ranking genes according to differential expressions between these two sets (using WRS), we found 21 genes that have a significantly lower expression in CB at FDR = 0.05 (genes and p-values reported in Differentially expressed genes CB vs. *BPx* in S1 Text).

Finally, in Metabric [23] the authors also report mutation status for 173 genes. We found (using a hypergeometric test) that mutations in *GATA3* have a lower frequency in the *BPx* class at FDR = 0.05, which is depleted at p-value = 0.003 (52/544 in *BPx*, 194/1360 in *GPx*). *GATA3* is one of three genes (the other two are *TP53* and *PIK3CA*) that were reported to have somatic mutations at more than 10% incidence across all breast cancers [24]. There is disagreement, however, about the role of *GATA3*, with some studies suggesting that *GATA3* functionality acts to inhibit, and other studies suggesting that it acts to promote, the development, growth, and/or spread of breast cancer. The second most significant gene with mutations observed, in terms of differences between *BPx* and *GPx*, is *MAP3K1* (50/544 in *BPx* and 173/1360 in *GPx*, p-value = 0.017). Both *GATA3* and *MAP3K1* are known to have important roles in breast cancer [24–27]. In the Metabric cohort there were ten patients observed with somatic mutations in both *GATA3* and *MAP3K1*. All ten patients were *BPx* (hypergeometric p-value = 3.41 × 10^−6^, N = 1904, B = 10, n = 544, b = 10). Moreover, nine of them were LumA and Her2+ *BPx* patients (hypergeometric p-value = 1 × 10^−7^, N = 1904, B = 10, n = 253, b = 9).

### Limitations and future directions

In this work we applied feature selection techniques to improve a prognostic immune related signature previously suggested in [15]. We obtained a classifier that uses 193 genes and yields a log rank p-value < 2 × 10^−4^ on test data. As we noted above, using different training validation set splits may yield different results. We ran the process with ten different splits (see Table 3). We observed 11 genes in the intersection of the 10 sets obtained in this manner. Observing such an intersection at random, under a uniform/independent null model, is highly significant (p-value < 10^−100^).

**Table 3:** Results for ten different splits.

	# genes	Validation accuracy	Log-rank p-value
1	91	93.4%	0.001
2	192	90.1%	0.0007
3	53	93.5%	0.0004
4	134	93.0%	0.04
5	109	93.4%	0.001
6	52	91.2%	0.004
7	193	93.1%	0.05
8	64	93.5%	0.02
9	68	93.0%	0.004
10	88	91.3%	0.003

It is important to note that the survival data used in this paper is overall survival data. In particular, our findings do not imply a signature that would be indicative of the response to any particular therapy.

To develop classifiers driven by survival data, one needs to use validation data for which feature values and survival information are available. The task of optimizing a classifier using quantitative features requires, and merits, much deeper investigation, in a broader context. The methods described in [28] address a similar question in the context of class discovery driven by a one-dimensional quantitative feature. That work does not, however, address survival to any extent.

**Fig 7:**
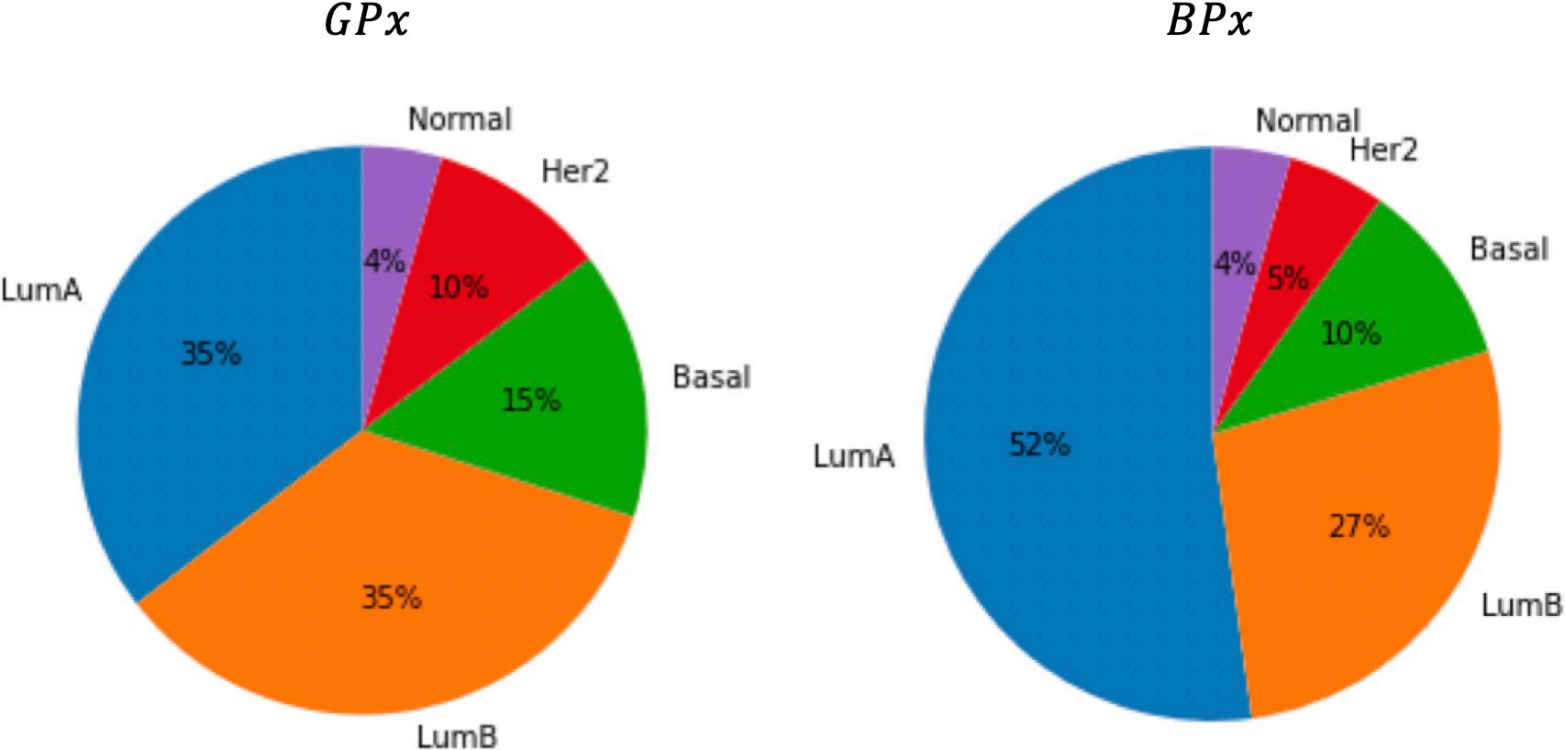
Pie charts of the PAM50 typing in the two different prognostic classes: *GPx* and *BPx*.

It is also interesting to examine the classification obtained by the Naive Bayes classifier on the test samples. Fig 7 depicts the distribution of PAM50 subtypes in the two classes, *BPx* and *GPx*. Note that although the PAM50 partition is not independent of the class (*χ*^2^ p-value = 0.002), neither prognostic subset is significantly aligned with a PAM50 subtype.

One obvious future research direction is the development of even more efficient prognostic signatures for breast cancer. A related task would be to include other types of measurement (copy number measurement, methylation, miRNA profiling etc.) to improve the predictive power.

Can the combination of Tumor Infiltrating Lymphocyte (TIL) levels with the expression signature lead to an even more significant prognostic indication? According to [15] and to other studies, TIL levels can be important in this context. An extension of our study would be to see how we can combine the expression-based immune signature with information about TIL levels.

The development of expression-based signatures calls for using sparse feature selection techniques. The literature addresses sparse feature selection in a variety of machine learning contexts. Questions related to which of these methods work for survival analysis is a topic for further research. In this study we explored several heuristic approaches. Some of them can be further generalized. Moreover, the direct use of survival data to drive feature selection and the development of methods for doing so will require further research and development.

## Conclusions

In this work we applied feature selection techniques to produce a more efficient signature that follows the one proposed by Tekpli et al. [15] and that can thereby define a subtype of breast cancer. We propose two feature selection approaches. The first is a variant of standard wrapper methods. Prognostic value is only assessed at the test stage. In the second one, we make a first attempt to use survival data as part of the learning process in developing a classifier. We find the combination of known subtyping with a survival driven approach to be an interesting future line of research.

The immune related signature suggested in Tekpli et al. [15] is likely to lead to better understanding and possibly better typing and diagnosis of breast cancer. Efficient approaches to subtyping, such as suggested in this current work, will further accelerate this process.

## Supporting information

S1 Text Genes lists and Confounding factors analysis. (PDF)
