# Supplementary material for: Efficient gene expression signature for a breast cancer immuno-subtype
Source: PLoS One. 2021 Jan 12;16(1):e0245215. doi: 10.1371/journal.pone.0245215 (PMC7802952; doi:10.1371/journal.pone.0245215)
Supplement: S1 Text — (PDF) [file pone.0245215.s001.pdf]

<u>Point A gene list</u>:
The following genes were selected according to the highest validation accuracy as described in the main text.

SLC27A, SPRR1A, CHRNA6, NECTIN3, DENND2D, SDHAF3, RUNX1T1, TMEM30B, CYTH2, NPHS2, IL7R, AKAP12, FBXO3, GFOD2, CD27, CSRP2, NEK9, CXCL9, DGKB, PSD4, TBC1D12, GCM1, C1GALT1C1, LCP2, ADSS, BCL11A, PHF20, BCL11B, PKD2L1, SMAD1, NPEPPS, PRG2, DSCR3, CSDC2, KDM5D, PAMR1, GAB1, ZNF365, RBM15B, NIPBL, CD79B, ZGPAT, SKIV2L, SLC35A1, ARHGAP5, APOLD1, PSMD6, CRYBG2, NOL8, CEP164, GPX3, PPARGC1A, SRC, ZNF177, CHST11, HOPX, RPL35A, DDX50, TDO2, PGAM1, ACAP1, KIAA1456, CCDC121, FAM189A2, ZNF226, RHOQ, SEC24D, SLAMF7, KCNAB1, CYP19A1, ITGAE, TMCO6, UGP2, HAAO, GAST, FAM234B, HMOX1, HIC2, ADAMTSL2, LTA, PPP2R2B, ADAMTS7, ZFAND5, APOOL, ABL1, COLQ, F13A1, TGFB3, EIF2B1, HTR2B, RASAL2, KIR3DL3, ABCA2, ABCG4, RAD54L, FGD2, BATF3, FASTKD3, BGN, BIRC3, GOLGB1, PTPN22, CCKBR, AQP4, RPP14, PSMD12, CD247, PDIA6, ODF1, DAZL, DNAJB9, FAM53B, DIO2, IPCEF1, MITF, SPACA9, FST, BASP1, TRIM10, GSPT2, ZNF280D, ZC4H2, GABRA3, KCNQ3, BCAT1, AQP7, GFM1, GRP, NPLOC4, ICAM3, AMPD1, PYHIN1, UGT2B15, PPP4R3A, CNPY4, XRCC4, INHBA, SEMA4A, CD3G, TNFRSF1A, COL4A5, MFAP3L, PDZD8, GNAT1, ELOVL1, CXCL10, MR1, STAG3, ARMT1, RARA, AZGP1, LSP1, SLC35E3, DPYS, C7orf69, CLCN2, DNAJC11, PDLIM3, KCNJ16, MINDY2, CDH19, TRIM29, SLC38A3, NEIL3, TRIM17, ACTR3, C17orf80, PEX3, SHOC2, FAH, NPC1, CHSY1, RAMP3, WASF3, STRAP, MBNL2, CXorf57, NES, DNMT3B, NIPAL2, TMX4, HAUS7, HIC1, CYP2R1, PIK3IP1, LILRB4, IL23A, PSMC3IP, NCAM1, ANK2, KSR1, GNAI1, TMCC2, CMTM6, TNFSF14, GRIK1, TXK, CDK5RAP3, OPA1, IL17A, HYOU1, PLEKHB2, GRM1, SDHAF1, TNS2, BTG2, FLOT1, EGLN1, MOGS, MEOX1, SMPD2, ELK3, TMEM206, DCK, PIM2, NDNF, FLOT2, HPRT1, RPA1, BICRA, APC, FBXL15, PTPRM, VDAC3, IL13RA2, SEC63, DCP1A, CD8B, GPR132, WDR1, SIRPG, C3orf14, TOR1AIP2, USP6NL, SCIN, MTCH1, CDK6, ENDOU, HOXC8, MYOM2, FIBP, ATP8B2, CNNM2, TPP2, TUBB2A, C7orf25, PFDN1, HTR3A, RXFP3, RPL18, PRRG1, CCL22, EFCAB6, NMNAT2, CCL17, CALM3, MAP4K1, TAP2, STRN3, SLC41A3, CXADR, PILRA, GPR25, GALNT2, PEX6, EBF2, USP6, CD200, PPP4R2, GRPR, CCNG1, CKAP4, BNC1, FAAP20, PPY, PIWIL2, ZNF136, WDR70, SORBS1, CRCP, HLA-DOA, UPK3A, LCK, TCEAL9, KIR2DL4, ZBTB32, LCOR, PDE11A, PTGES, CAPN9, NPAS1, SCGB1D2, M6PR, NEK1, TNNT3, POLR2C, SLC30A3, SALL1, CTNNB1, USP16, CACNA1G, G0S2, C6orf10, TPH1, EXTL1, DND1, APOB, CYTIP, TMEM140, PRSS1, COL8A1, BACH2, FOXE3, HIF3A, UQCRC2, FAIM, ST6GAL1, FGGY, PRKCG, LIG3, AQP9, SLC7A4, KCNA1, UBTF, EPN2, TNFRSF8, CHRNA7, DFFB, C1orf105, ATP12A, CPPED1, TNFRSF11B, CBWD5, MAGEA4, FASTKD2, ERAP2, IGLL1, CDKL1, GLRX, EXOC6B, PTPN6, GTF3C2, RAPGEF5, IL26, MED7, POLR2J2, ME3, RB1, SERPINI2, SYNE1, TMEM5, EHF, IL12RB1, SNX11, BIN1, GRK5, NFS1, PLPPR1, PBX3, FARP2, PLCL2, NGF, TEDC2, DCHS1, TOMM7, WDR26, PIP4K2A, SCAF8, HNRNPA1, IL1RAPL2, ZNF107, HSD17B1, MAU2, FBXO2, CTDSPL, ATF6, CD2, CNDP2, TPSG1, CD93, PTPRT, COL4A3, NUMA1, RGS3, ATP5O, HFE, CELF3, WNT3, ORC1, ASPN, HPS4, ALDH1A1, FABP3, NCOA1, CDK4, PYGO2, P2RX5, CYP11A1, PPP2R1B, RPL31, NKX3-2, H6PD, TP53BP1, CYB5R3, CCDC25, ZNRF4, SEMA3E, MYO7A, CDH17, GLUD2, ATF3, PIGC, PRKCH, TMEM39B, RBM10, HPX, DENND1A, MTMR12, SCN2B, PARP8, ZDHHC17, LDLRAP1, HDGFL3, RETREG1, TAPBP, ZNF362, MYH7, INTS6, SLC2A9, SLC34A2, VIPR2, PRKCB, TRIM15, SCD5, EIF1B, TUBA1A, RMND5A, WDR45, PTPRA, ETAA1, RPSA, UBXN7, EBP, ARHGAP10, XYLT1, PRDM8, RAB11A, CRADD, CD38, PDCL3, CD8A, TNNC2, CYP7A1, BCAN, LYPD1, CCRL2, KEL, MBIP, ME2, PPM1A, TMEM2, TIPARP, MS4A4A, FA2H, LBH, KLF2, SLAMF1, EXOC3, SIGLEC15, PRDM9, ZXDC, SLC6A4, TTR, EPHX2, ZNF350, KDR, CETP, APAF1, MANEA, CTDSP1, SERPINE2, DPP6, ISCU, ARHGEF12, AIDA, MBP, HP, SLC45A2, P2RY14, PI3, DNMT3A, ATG13, ST3GAL2, KCTD15, MAGEA8, DUSP10, ACTL6B, HYAL3, IL4R, LPGAT1, TRIOBP, MCOLN1, ARHGAP35, TUBB2B, MME, CYB5A, GTF2A2, MOXD1, KLRG1, KCNA5, TNKS2, TMEM127, DUSP3, PTPN14, PRAME, PRCP, ATP2A2, MS4A2, ARC, SCN3A, DIP2C, CASP3, KIAA0753, FHL5, WNT16, NECAB3, KRT2, GAS6, ITIH5, ABCD4, FRMD4A, NFE2L1, CHST15, IL20RB, PYGL, CFHR4, LAG3, IL18R1, TSFM, C5orf42, CBFA2T3, CTRL, PDCD1

<u>Point B gene list</u>:
The following genes were selected according to the lowest survival p-value on the validation set as described in the main text.

ACP1, PBX3, CD93, ZNF365, LYPLA2, DCP1A, RASAL2, RPL31, TNS1, NUP160, PMS2, ACTR3, NFS1, NCOA1, PRDM8, AQP7, BTG2, CLK4, RARA, FST, MITF, CDK5RAP3, KCNS3, ENDOU, ATP5O, PIP4K2A, TNFSF14, CXorf57, PIGC, IL26, STIM1, SREK1, ZFAND5, CAST, C1GALT1C1, TUBD1, RAPGEF5, CD38, TOR3A, FBXO3, EIF4A3, HNRNPA1, HTR2B, RBM41, CD27, KCNK1, XPO6, SNRK, BAZ1B, SLC2A9, NPC1, SRC, PDZD8, ARHGEF12, PRF1, RHOQ, GRP, PAQR3, MAGEA4, LILRB4, CYTIP, HDGFL3, UQCRC2, ARHGAP10, ITGB1, C12orf4, PRKCH, BCL11A, PLPPR1, PDCD1, SLC9A1, CCNG1, PTPN22, NUMA1, RPL35A, FOXN2, ALDH1A1, LCK, BCL2L1, KDR, ASPA, TUBB2B, TRIM10, HECW1, TSFM, CYP2R1, CDKL1, COL4A5, KCTD15, MAL, AZGP1, RUNX1T1, CMTM6, UBTF, P2RX5, CCL5, GFM1, EFCAB6, HLA-DOB, CELF3, HEBP1, LRRC17, B3GNTL1, CPVL, BGN, RAB11A, CXCL9, CPPED1, MSN, TOMM7, GPR132, CCNJL, CDK6, NECAB3, CTRL, MDM2, STAG3, FRMD4A, CD2, IL7R, DRAP1, PTPRM, LAG3, KSR1, LCOR, LIG3, C3orf14, ARHGEF11, PYGO2, AIDA, NEK3, RAD51C, COL4A3, CYTH2, CTNNB1, G0S2, AKAP12, TP53BP1, MID1, DUSP3, PYHIN1, TMEM2, TRAIP, HLA-DOA, RAPGEF3, SEC24D, CHSY1, PLCL2, HIC2, MPZL2, CYP39A1, BIRC3, PRSS3, TRIOBP, BNC1, NFE2L1, SLAMF7, MTMR12, SNCG, TNKS2, IGLL1, ME3, CHST11, ZNF107, DNAJB9, SEC63, HPS4, ATG13, HAUS7, EIF4E2, SNX2, TMEM140, LAGE3, P2RY14, PRAME, GLI2, ATP11A, TMEM30B, LYPD1, FAM53B, SH3GL1, GPX3, MOGS, POP7, KIR2DL4, FGD2, KLRG1, GOLM1, COL8A1, SLC35E3, RAB35, ZNF350, NCOR1

<u>Differentially expressed genes CB vs. BPx</u>:
The following genes have significantly lower expression in CB compared to BPx (using WRS) at FDR=0.05.

Gene	p-value
CD2	0.00003
HTR2B	0.00005
CPVL	0.00006
ASPA	0.00007
IL7R	0.00008
ALDH1A1	0.00014
CCL5	0.00030
TMEM140	0.00039
AQP7	0.00058
CD93	0.00113
FOXN2	0.00144
P2RY14	0.00156
PYHIN1	0.00178
AKAP12	0.00187
CD27	0.00209
BIRC3	0.00267
RAPGEF3	0.00340
TUBB2B	0.00399
HLA-DOA	0.00434
PLCL2	0.00512
GPX3	0.00513

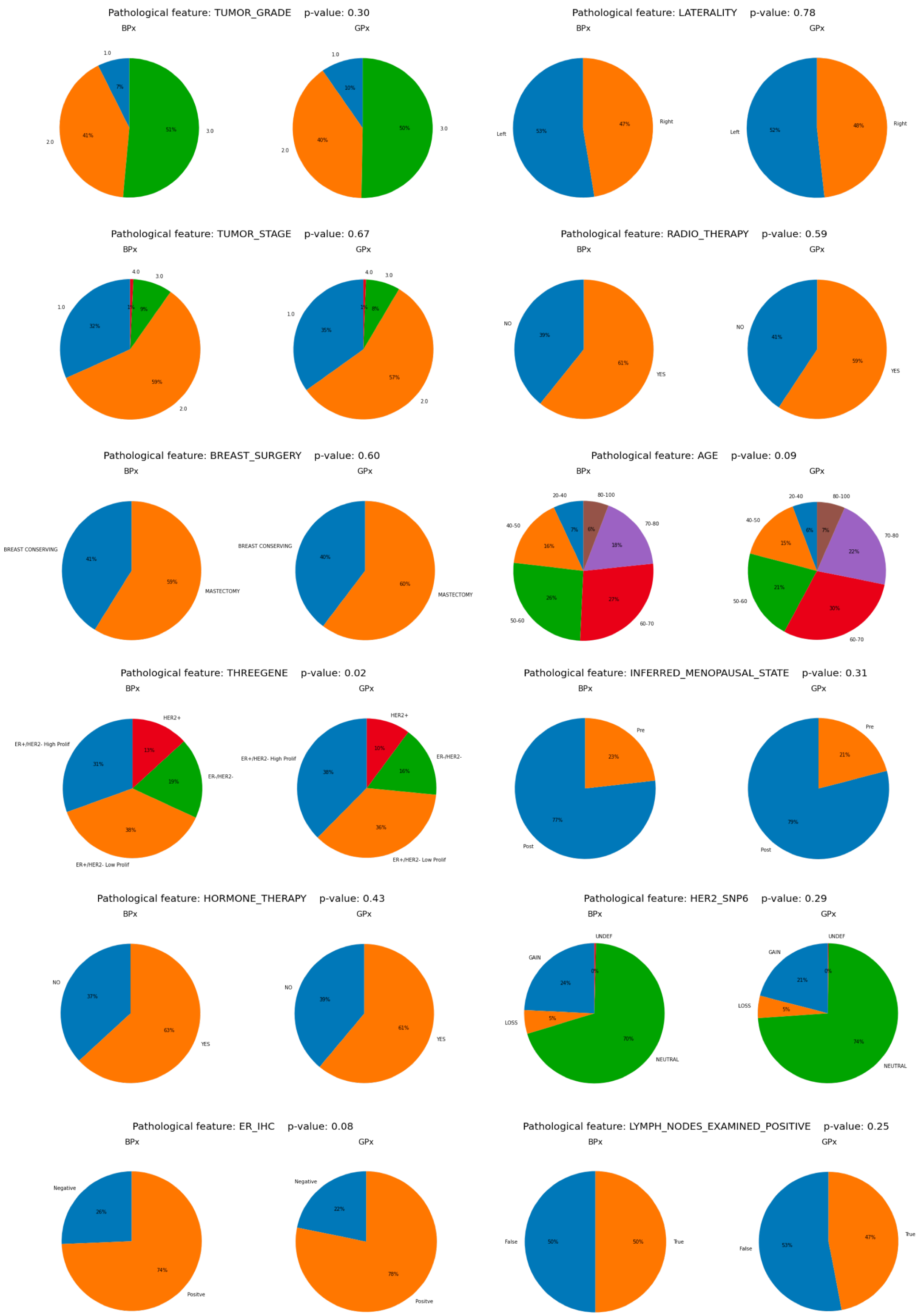

S1 Fig 1: **Confounding factors distribution BPx vs. GPx.** Each pie chart represents the distribution of a pathological feature in our two classes BPx and GPx. Data from the Metabric cohort. The p-value was calculated using chi-square test with the no difference null hypothesis. At FDR=0.05 we cannot reject the null hypothesis for any of the features.
